# Biological Activity of Monoterpene-Based Scaffolds: A Natural Toolbox for Drug Discovery

**DOI:** 10.3390/molecules30071480

**Published:** 2025-03-27

**Authors:** Jarosław Mołdoch, Monika Agacka-Mołdoch, Grzegorz Jóźwiak, Karolina Wojtunik-Kulesza

**Affiliations:** 1 Department of Biochemistry and Crop Quality, Institute of Soil Science and Plant Cultivation, State Research Institute, 24-100 Puławy, Poland; jmoldoch@iung.pulawy.pl; 2 Department of Plant Breeding and Biotechnology, Institute of Soil Science and Plant Cultivation, State Research Institute, Czartoryskich 8, 24-100 Puławy, Poland; magacka@iung.pulawy.pl; 3 Department of Inorganic Chemistry, Medical University of Lublin, Chodźki 4a, 20-093 Lublin, Poland; grzegorz.jozwiak@umlub.pl

**Keywords:** monoterpenes, molecular scaffold, carvacrol, carvone, citral, menthol, *β*-pinene, thymol, verbenone, menthone

## Abstract

One of the most common strategies used in drug design is the molecular scaffold approach, which combines traditional medicine based on natural active compounds derived from plants with modern synthetic drug development. Designing new compounds based on natural skeletons enables extensive modifications of both bioavailability and biological activity. An excellent example of a natural molecular scaffold is the monoterpenes group, which serves as a core structure for building more complex molecules by attaching various chemical groups. Their ability to interact with biological targets, combined with structural versatility, makes them promising molecular scaffolds in pharmaceutical research and green chemistry applications. This review paper focuses on selected monoterpenes (carvacrol, carvone, citral, menthol, menthone, *β*-pinene, thymol, and verbenone), which are frequently used as molecular scaffolds. The newly designed derivatives exhibit various biological activities, including anticancer, antibacterial, antiviral, neuroprotective, and many others.

## 1. Introduction

Drug design poses a significant challenge for modern medicine. It is well known that new active compounds, which form the basis of commonly used drugs, should possess several crucial features such as effectiveness, safety, and commercial viability. Active compounds must demonstrate high efficacy at low doses, be soluble in both fats and water to influence absorption and distribution, and exhibit high selectivity by preferentially binding to a specific biological target to minimize side effects and avoid toxicity or mutagenicity, among other characteristics [1]. Identifying compounds that meet these criteria is highly complex. Considering the extensive series of studies (in vitro, in silico, in vivo) and clinical trials required, the process of bringing a new drug to market takes many years and involves the collaboration of numerous scientists.

A significant facilitation in drug design is the generation of new compounds using molecular scaffolds. The concept of the molecular scaffold is one of the most widely applied approaches in medicinal chemistry. The term ‘molecular scaffold’ is defined as the core structure or framework of a molecule that serves as a central template onto which functional groups or side chains can be attached to create derivatives with varying biological activities. This concept was first introduced by Bemis and Murcko (‘BM scaffold’), who proposed a hierarchical molecular organization scheme by dividing small molecules into R-groups, linkers, and frameworks [2,3]. The scaffold represents the central molecular framework used in drug design, identified during virtual screening, or utilized as a platform for synthetic modifications. In a set of related compounds, the scaffold can be determined by identifying the largest shared substructure; however, several alternative methods for defining scaffolds also exist. As with many scientific concepts, BM scaffolds may generate inaccuracies. Different BM scaffolds can represent highly similar structures that differ only in minor aspects, such as the position of a single heteroatom or slight changes in bond order [3]. Despite these limitations, the BM approach remains the most widely used method for defining molecular scaffolds. These structures serve as a starting point for advanced modifications aimed at obtaining compounds with specific biological activities, improved safety profiles, and enhanced bioavailability. Many molecular scaffolds are derived from natural products, as plants are an inexhaustible source of bioactive compounds with high structural diversity and safety. A significant proportion of currently available drugs are based on natural products or their derivatives. For centuries, substances of natural origin have been used to produce medicines for various diseases. Natural compounds have played a pivotal role in the development of pharmacy and the pharmaceutical industry, where synthetic drugs are often designed based on molecular scaffolds derived from nature. Examples of natural molecular scaffolds include coumarin, chromone, chalcone, aurone, caffeine, and many others [4,5].

Among natural compounds frequently used in the design of new active compounds are monoterpenes. These compounds belong to the largest group of secondary plant metabolites, namely terpenes, which exhibit diverse structures and biological activities. Monoterpenes are often highlighted as a significant group of compounds due to their wide spectrum of biological activity, good bioavailability, and capacity to cross the blood–brain barrier (BBB) [6]. Low-molecular-weight and volatile isoprenoids are widely present in plants that have adapted to survive under harsh environmental conditions, such as intense UV radiation, high soil salinity, and drought. These challenging conditions are typical of the Mediterranean macchia, an ecosystem rich in plants known for their high production of volatile oils. Examples include *Rosmarinus officinalis* L. (rosemary), *Juniperus oxycedrus* L., *Erica scoparia* L., and many others. As an alternative photoprotective strategy, these plants synthesize isoprenoids, including monoterpenes [7]. A growing area of research focuses on traditional plant-based medicine, as numerous bioactive compounds derived from plants are already utilized in modern pharmaceuticals. Among these, terpenes play a significant role. These secondary metabolites exhibit a wide range of biological activities, including antioxidant properties, enzyme inhibition (such as acetylcholinesterase—AChE, amylase, and glucosidase), antifungal effects, hepatoprotection, and sedative actions [8,9]. The low molecular weight, good bioavailability, and interesting biological activities of monoterpenes form the basis for their use as molecular scaffolds.

**Figure 1:**
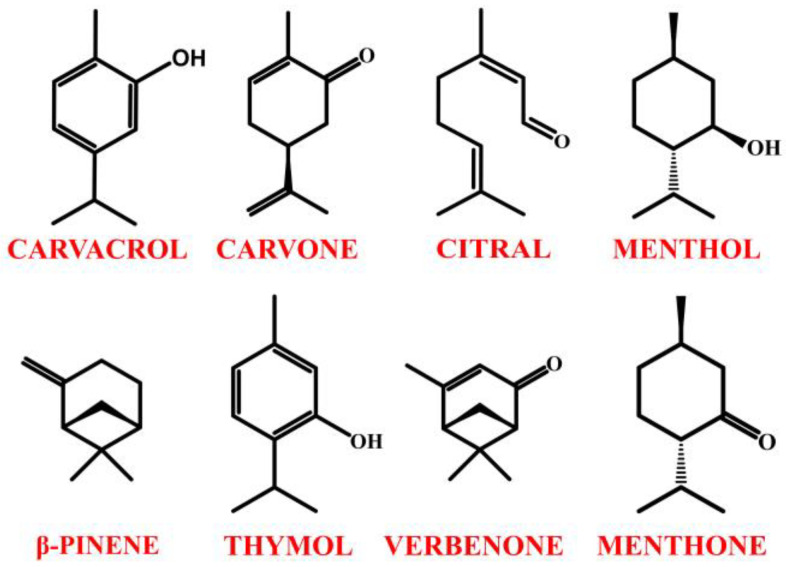
Structures of monoterpenes presented in the paper as molecular scaffolds.

Advancements in the total synthesis of natural products have significantly enhanced the accessibility and study of diverse biologically active molecules, driving innovation across multiple fields, including agriculture, materials science, nanotechnology, biology, medicine, and pharmacology [10,11]. Among natural product scaffolds, monoterpenes—C_10_ hydrocarbons derived from isoprene units—have emerged as valuable synthetic building blocks due to their structural complexity, abundance, and intrinsic biological activity. Their characteristic cyclic or acyclic frameworks often feature multiple functional groups and chiral centers, making them ideal precursors for stereoselective transformations [12,13]. Leveraging monoterpenes in synthesis enables the creation of novel bioactive compounds with improved pharmacological properties, contributing to drug discovery, agrochemical development, and material science advancements. Their versatile reactivity and chirality offer unique opportunities to design and optimize new therapeutic agents with enhanced selectivity and potency. The presented review article aims to characterize selected monoterpenes (carvacrol, carvone, citral, menthol, *β*-pinene, thymol, verbenone, menthone) (Figure 1) in terms of their molecular scaffold applications. The paper focuses on specific compound derivatives, which were analyzed for their potential pharmaceutical applications.

This review provides a comprehensive, up-to-date perspective on how monoterpene-based scaffolds can be optimized for therapeutic applications. It will be particularly beneficial for researchers seeking insights into the structure–activity relationships of monoterpene derivatives and their potential for future drug development. This paper systematically examines how structural modifications impact biological activities such as anticancer, antibacterial, antiviral, and neuroprotective effects. The review strategically focuses on a curated selection (carvacrole, carvone, citral, menthol, menthone, *β*-pinene, thymol, and verbenone), providing a detailed discussion on their scaffold-based derivatives and pharmacological potential.

## 2. Carvacrol

### 2.1. Biological Activity

Carvacrol exhibits a broad spectrum of biological activities, including antimicrobial activity, making it suitable for use as a natural preservative and anti-infective agent [14,15,16]. Its anticancer and antimutagenic properties result from its ability to inhibit cancer cell proliferation and protect DNA from mutations [17,18,19,20]. Studies have also demonstrated its anti-inflammatory effects, which may be applied in the treatment of gastrointestinal inflammation and rheumatic diseases [21]. Carvacrol possesses antispasmodic and analgesic properties, making it a potential natural remedy for relieving muscle pain and spasms [22,23]. Additionally, carvacrol displays antiparasitic activity, suggesting its potential use in the treatment of parasitic diseases [24,25,26], along with an antiplatelet effect that may help reduce the risk of blood clots and promote cardiovascular health [27,28]. Another significant property is its ability to inhibit acetylcholinesterase (AChE), which may be relevant in the treatment of neurodegenerative diseases, such as Alzheimer’s disease [29,30,31]. Its anti-elastase activity indicates potential applications in cosmetology and dermatology, particularly in anti-aging skin preparations [32]. Furthermore, its insecticidal properties suggest its potential use in plant protection and pest elimination [33,34,35].

### 2.2. Biological Activities of Carvacrol Derivatives

**Figure 2:**
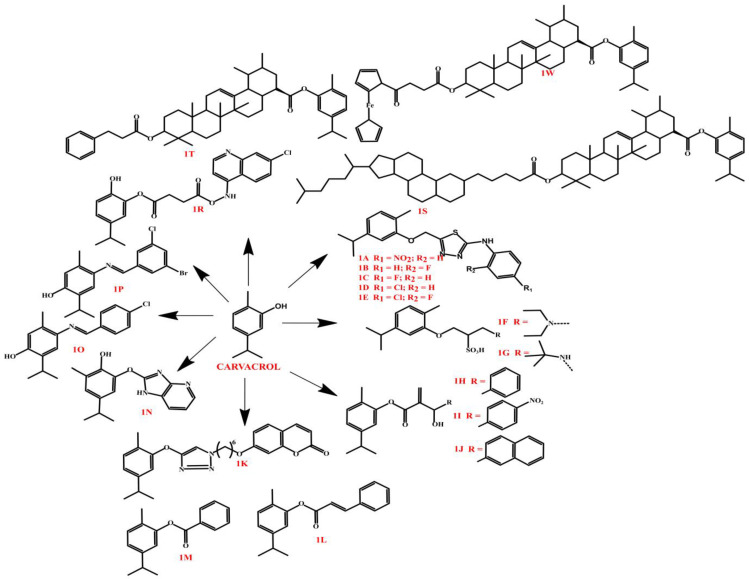
Carvacrol derivatives.

**Figure 3:**
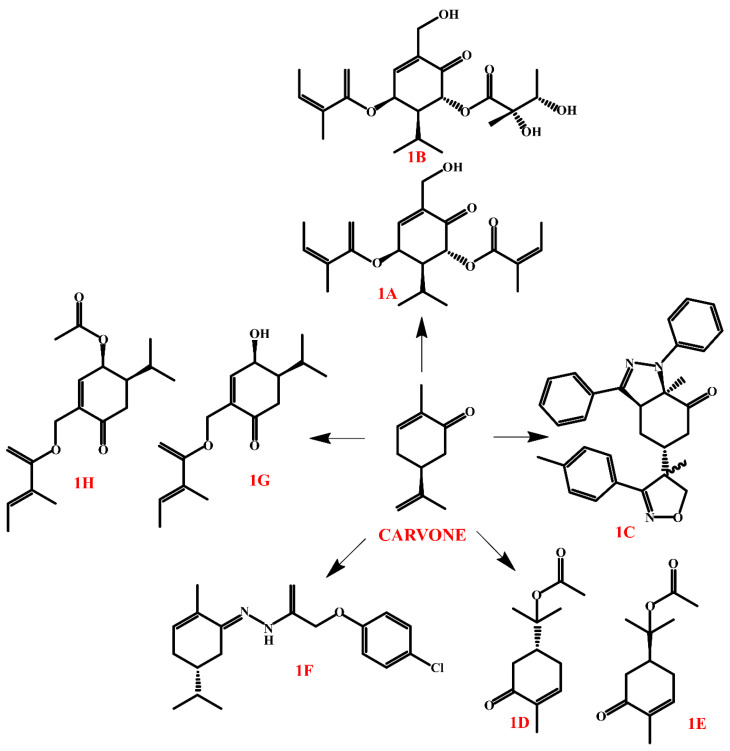
Carvone derivatives.

Carvacrol derivatives are an interesting group of chemical compounds with a broad spectrum of biological activities, including anticancer, antimicrobial, and inhibitory properties against key metabolic enzymes. Structural modifications of carvacrol, such as the introduction of thiosemicarbazide, sulfonic, coumarinic, or benzimidazole groupings, significantly improve its biological activity and may form the basis for the development of new drugs and therapeutic agents. Numerous studies have shown that carvacrol derivatives have potential applications in the treatment of neurodegenerative diseases, cancers, and bacterial infections, especially in the context of pathogens resistant to classical antibiotics. The newly synthesized carvacrol derivatives, which include thiosemicarbazides **1A**–**1E** (Figure 2) and 1,3,4-thiadiazol-2-amine derivatives, have shown strong inhibitory properties against key metabolic enzymes, making them potential candidates for therapeutic applications. Enzyme assays showed that all compounds tested effectively inhibited human carbonic anhydrases I and II (hCA I and hCA II), acetylcholinesterase (AChE), and butyrylcholinesterase (BChE), with activity superior to standard inhibitors. Further, molecular docking analysis confirmed the high specificity and strong affinity of the tested carvacrol derivatives for the enzymes, increasing their potential therapeutic value. The significant inhibition of AChE and BChE points to the possibility of using these compounds in the treatment of neurodegenerative diseases, such as Alzheimer’s disease, through a mechanism of inhibition of acetylcholine-degrading enzymes. Inhibition of carbonic anhydrases, on the other hand, suggests the possibility of using these derivatives in the treatment of glaucoma, epilepsy, or cancer. The results obtained confirm that the newly synthesized carvacrol derivatives may represent promising enzyme inhibitors with high therapeutic potential [36]. The newly developed carvacrol derivatives, comprising eight 2-aminothiols and three sulfonic acid derivatives, showed potent inhibitory activity against key metabolic enzymes. Their activity was assessed in terms of inhibition of the isoenzymes human carbonic anhydrase I and II (hCA I and hCA II), acetylcholinesterase (AChE), butyrylcholinesterase (BChE), and *α*-glucosidase. Of the compounds tested, sulfonic acid derivatives **1F** and **1G** (Figure 2) showed the highest efficacy against carbonic anhydrase isoenzymes, suggesting their potential use in the treatment of metabolic disorders. The potent inhibitory activity of AChE and BChE indicates the potential use of these derivatives in the treatment of neurodegenerative diseases, while the ability to inhibit *α*-glucosidase suggests potential use in the treatment of type 2 diabetes by reducing carbohydrate absorption [37]. In terms of anticancer activity, the **1H**–**1J** (Figure 2) derivatives showed significantly higher cytotoxicity than carvacrol [38]. Of these, **1J** showed the highest selectivity, suggesting its potential for further preclinical studies. In addition, derivative **1I**, which is a hybrid of coumarin and carvacrol, showed potent cytotoxic activity against MCF-7, PC-3, and HT-29 cancer lines. Structural modification of carvacrol to link to the coumarin system may have significantly improved its biological activity [34].

In studies on antimicrobial activity (compounds **1K**–**1N**, Figure 2), derivative **1N**, a hybrid of benzimidazole and carvacrol, showed potent activity against *S. aureus*, *E. coli*, *S. pyogenus*, and *P. aeruginosa* [36,39]. Fluoroalkyl and alkyl substitutions (compounds **1O** and **1P**) significantly increased the antibacterial efficacy of these derivatives. Derivative **1R** (Figure 2), which is an ester of carvacrol with a 4-aminoquinoline grouping, showed even higher antibacterial activity, and its efficacy was superior to that of carvacrol alone. In addition, derivatives **1S**–**1W**, which are hybrids of ursolic acid and carvacrol, showed significant activity against *Proteus vulgaris* and *Proteus mirabilis* strains, indicating the potential of these derivatives in the treatment of bacterial infections, especially in the context of pathogens resistant to classical antibiotics [40,41]. In summary, the most active carvacrol derivatives have clear potential in both enzymatic, anticancer, and antimicrobial applications. In particular, derivatives **1A**–**1E** and **1F**–**1G** show strong inhibitory activity against metabolic enzymes, derivatives **1H**–**1J** stand out for their potent anticancer activity, while derivatives **1M** and **1O** have high antimicrobial efficacy, suggesting their possible use in the development of new antibiotics.

## 3. Carvone

### 3.1. Biological Activity

Carvone exhibits a wide range of biological activities, making it a promising compound in the context of potential therapeutic applications. Its antimicrobial properties include antimicrobial and antifungal activities, indicating its potential use in the treatment of infections [42,43,44]. In addition, it exhibits strong antispasmodic effects, which may be important in the treatment of gastrointestinal and respiratory disorders by inhibiting smooth muscle contractions [43]. Carvone also has anti-inflammatory properties, reducing the production of inflammatory markers, suggesting its potential use in the treatment of chronic inflammatory diseases [45]. In addition, it exhibits antioxidant activity by neutralizing free radicals and protecting cells from oxidative stress [43]. In terms of effects on the nervous system, carvone shows anticonvulsant properties, reducing the frequency and severity of epileptic seizures, as well as analgesic effects through modulation of the mechanisms responsible for pain perception [46]. In addition, it has potential antidiabetic properties, affecting the regulation of blood glucose levels, which may support diabetes therapy [47]. Its anti-anxiety and sedative effects suggest potential use in the treatment of neurological disorders, including anxiety [48]. It also has neuroprotective properties [49]. Preliminary studies also suggest the antitumor activity of carvone, showing cytotoxic activity against cancer cells, making it an interesting candidate for further research in oncology [50]. In addition, it exhibits antiparasitic activity, which can be used to eliminate certain parasites [51]. Due to its diverse pharmacological properties, carvone represents a promising monoterpene with potential therapeutic relevance, but further research is required to understand its exact mechanisms of action and safety of use.

### 3.2. Biological Activities of Carvone Derivatives

Carvone derivatives exhibit a broad spectrum of biological activities including antibacterial, anticancer, anti-inflammatory, analgesic, anticonvulsant, and insecticidal activities, making them promising candidates for pharmaceutical and agrochemical applications. Carvone and its derivatives have strong antioxidant properties, as confirmed by DPPH and ABTS free radical scavenging tests. *Mentha spicata* essential oil, which has a high carvone content, has shown significant antioxidant activity, reducing oxidative stress in neuronal cells and inhibiting the activity of pro-oxidant enzymes [51].

In the context of antimicrobial activity, (+)-carvone has shown moderate activity against *Escherichia coli*, *Salmonella typhimurium*, and *Staphylococcus aureus*. However, carvone derivatives, such as compounds **1A** and **1B** (Figure 3), have shown much stronger activity against *Mycobacterium aurum* and *Mycobacterium bovis* BCG. The essential oil of *Pluchea carolinensis*, rich in carvone derivatives, strongly inhibited the growth of *Bacillus cereus* and *Staphylococcus aureus*, indicating the potential of these compounds as natural antimicrobial agents [42,51].

The **1C** (Figure 2) derivative, which is a pyrazole–isoxasole hybrid compound, showed low anticancer activity (IC_50_ ≥ 100 μM) against selected cells (HT-1080, MCF-7, A549, and MDA-MB-231). The mechanism of action of **1C** was confirmed by molecular docking studies, where it showed a strong affinity for the BCL-2 protein, a key regulator of apoptosis in cancer cells. BCL-2 is responsible for inhibiting programmed cell death, and its overexpression is often associated with cancer resistance to treatment. The inhibition of this pathway by **1C** suggests that this compound may act as a BCL-2 inhibitor, leading to the induction of apoptosis in cancer cells [52].

The anti-inflammatory activity of carvone derivatives is manifested by reducing the expression of pro-inflammatory cytokines, such as TNF-*α*, IL-1*β*, and IL-6, and inhibiting the activity of the transcription factor NF-κB, which effectively reduces inflammatory responses. Derivatives **1D** and **1E** (Figure 3), which were most effective, inhibited iNOS expression and NO production, suggesting their potential use in the treatment of inflammatory diseases. These derivatives also showed the highest predicted activity against Sirtuin-1 (SIRT1), an enzyme involved in the regulation of inflammatory response and metabolism [53].

Hydrazone **1F** (Figure 3) showed the strongest anticonvulsant and analgesic activity among the (−)-carvone derivatives tested. Its antiepileptic activity manifested itself as effective protection against both electroshock (MES)- and pentylentetrazole (PTZ)-induced seizures. Importantly, this effect was sustained over both short (1 h) and long (24 h) periods, suggesting the potential use of this compound in the treatment of epilepsy. In addition to its anticonvulsant effect, hydrazone **1F** showed the highest efficacy in capsaicin and allilothiocyanate (AITC)-induced pain models. Its analgesic mechanism was related to the modulation of TRPA1 and TRPV1 ion channels, which play a key role in pain perception. Its potent analgesic activity suggests that the compound may find use as an analgesic agent, particularly in the treatment of neuropathic pain. The high efficacy in both seizure inhibition and pain reduction makes hydrazone **1F** a promising candidate for further research on developing new epilepsy drugs and innovative analgesic therapies [46]. Carvone derivatives isolated from *Blumea mollis* show potent mosquitocidal and ovicidal activity against *Culex quinquefasciatus*. Compound **1G** (*4R,5S)-4-hydroxy-7-tigloxycarvanoacetone) and its derivative, compound **1H** (*4R,5S)-4-acetoxy-7-tigloxycarvanoacetone) (Figure 3), effectively eliminated mosquito larvae and eggs, with compound **1H** showing higher efficacy. The mechanism of action of these carvone derivatives included severe damage to the digestive system of the larvae and blocking acetylcholinesterase (AChE), a key enzyme of the insect nervous system. Importantly, toxicological tests confirmed their selectivity and lack of harm to non-target organisms, such as the fish *Poecilia reticulata*. Compound **1H**, as a more active carvone derivative, represents a promising natural insecticide with high selectivity and environmental safety [54]. In summary, carvone derivatives exhibit a wide range of biological properties including antioxidant, antimicrobial, anticancer, anti-inflammatory, analgesic, anticonvulsant, and insecticidal activities. Their therapeutic potential and application in plant protection make them promising compounds for further pharmacological and agrochemical research.

## 4. Citral

### 4.1. Biological Activity

Citral is a mixture of two aldehyde isomers, geranial and neral, which occur naturally in the essential oils of many citrus plants, such as lemongrass, lemon verbena, and citronella [55,56]. Due to its intense lemony aroma, it is widely used in the perfume and cosmetics industry and as a flavoring additive in foods [56]. It exhibits numerous biological properties, including antibacterial, antifungal, and antioxidant activity [57]. In vitro studies have confirmed its effectiveness in inhibiting the growth of both Gram-positive and Gram-negative bacteria, including *Staphylococcus aureus* and *Escherichia coli*, due to its ability to damage the bacterial cell membrane and inhibit the activity of key bacterial enzymes [57,58,59]. In addition to its antibacterial action, citral also exhibits antifungal properties, effectively reducing the growth of yeast-like fungi and molds [56,60]. In vivo studies have demonstrated its ability to reduce inflammation and oxidative stress, which may be relevant to therapeutic applications [61]. It has also been shown to have strong antiviral [62], anti-inflammatory [63,64], and antileishmaniasis activity [65,66], as well as the ability to inhibit cytokine activity [67], making it a potential candidate for use in the treatment of inflammatory and immunological diseases. In addition, it shows chemopreventive [68], allelopathic [33], and aldose reductase inhibitor [62] activity, which suggests its potential use in the treatment of diabetes and metabolic diseases. Citral also exhibits spasmolytic properties [69], antiadipogenic [70] and deterrent [71], which may find applications in both pharmacology and the cosmetics industry. In addition, studies have confirmed its antiparasitic activity [56,72], larvicide [71,73] and affecting cognitive functions [74], which could open up new possibilities in neurological therapy and the control of vector-borne diseases.

### 4.2. Biological Activities of Citral Derivatives

**Figure 4:**
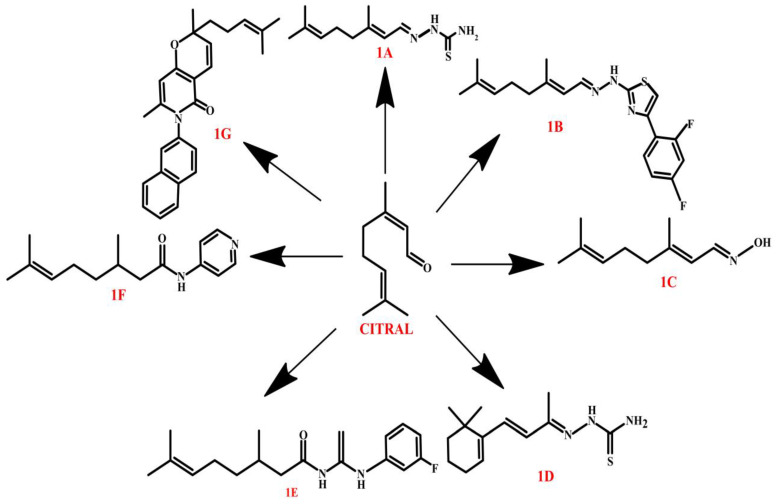
Citral derivatives.

Newly synthesized citral derivatives show a wide range of biological activities, including antifungal, antitumor, and anti-quorum sensing activities, making them promising candidates for further development as therapeutic and agrochemical agents. In terms of antifungal activity, numerous citral derivatives have shown high efficacy against plant pathogenic fungi, including *Colletotrichum gloeosprioides*, *Rhizoctonia solani*, *Phytophthora nicotianae* var*. nicotianae*, *Diplodia pinea*, *Colletotrichum acutatum*, and *Fusarium oxysporum f.* sp. *niveum*. Two particularly outstanding compounds, **1A** and **1B** (Figure 4), were superior in efficacy to tricyclazole, confirming their potential as modern fungicides [75]. In addition, the introduction of electronegative groups, such as fluorine, increased activity against all pathogens tested. Thiourea-grouped citral derivatives, such as **1D** and **1E** (Figure 4), showed high efficacy against *C. gloeosprioides*, with an EC_50_ in the range of 0.16–4.76 mg/L, outperforming standard fungicides including carbendazim. Their mechanism of action included an increase in the permeability of fungal cell membranes and damage to intracellular structures, which led to their elimination [76]. In the context of rice sheath rot control, the amide derivatives of citral showed significantly higher efficacy than citral, reaching EC_50_ values in the range of 9.50–27.12 mg/L. Compound **1F** (Figure 4) showed strong fungistatic activity by prolonging the growth retardation phase, inhibiting biomass and sclerotia germination. The mechanism of action included an increase in cell membrane permeability, resulting in destabilization of fungal metabolism, disruption of the tricarboxylic acid cycle, and reduction in the secretion of natural antifungal substances by *R. solani* [77].

In the antitumor area, the citral derivative **1G** (Figure 4) showed high efficacy against HepG2 hepatocellular carcinoma cells, outperforming both citral and cisplatin. The IC_50_ for 3 h was 5.3 μM, indicating a more potent cytotoxic effect than cisplatin (6.5 μM). The mechanism of action included cell cycle arrest in the S-phase and induction of apoptosis, as evidenced by an increase in the expression of the proapoptotic BAX protein and inhibition of the expression of the anti-apoptotic BCL2. In addition, **1G** inhibited key MAPK/ERK and PI3K/AKT proliferative pathways by decreasing the phosphorylation of PI3K, AKT, and ERK kinases, effectively blocking the growth of cancer cells [78]. Another important line of research was the anti-quorum sensing and antibiofilm activity of citral derivatives. Compound **1C** (Figure 4) showed the strongest anti-quorum sensing and antibiofilm activity, making it a promising candidate as a modern antimicrobial agent. **1C** effectively inhibited bacterial communication and blocked biofilm formation at subinhibitory concentrations, meaning that it eliminated biofilms without affecting bacterial growth. This mechanism was confirmed by RT-qPCR analysis, which showed decreased expression of quorum sensing genes, clearly indicating **1C**’s ability to interfere with bacterial communication. These results suggest that **1C** may represent an effective alternative to antibiotics, particularly in the context of combating multidrug resistance [79]. Similarly important studies were presented by Sepúlveda-Arias et al. [80] who, for the first time, evaluated the anti-inflammatory activity of epoxides obtained from Colombian plants’ (*Tagetes lucida*, *Cymbopogon citratus*, *Lippia alba*, and *Eucalyptus citriodora*) essential oils. Citral, the main component of *C. citratus*, carvone, the main component of *L. alba*, and their derivatives, 6,7-epoxycitral and 8,9-epoxycarvone, revealed high inhibitory potential toward the secretion of prostaglandins (PGE2) and NO associated with inflammation. Significant is also the fact that epoxides at the RNA level exhibited a low impact on iNOS expression, whereas they had a significant influence on COX-2 inhibition.

In summary, citral derivatives exhibit a broad spectrum of biological activities, including antifungal, anticancer, and anti-quorum sensing activities, making them promising candidates for further development. Their mechanisms of action include increasing the permeability of pathogen cell membranes, inhibiting proliferative pathways, and blocking bacterial communication, paving the way for their potential use in agriculture, medicine, and public health.

## 5. Menthol

### 5.1. Biological Activity

Menthol exhibits a broad spectrum of biological activities, including anti-inflammatory and analgesic effects on the digestive and respiratory systems, antimicrobial and antifungal properties, as well as the ability to improve alertness, thermal comfort, and athletic performance. Menthol exhibits potent anti-inflammatory effects, reducing levels of pro-inflammatory cytokines and inflammatory markers and inhibiting enzymes that regulate the inflammatory response. In vitro and in vivo studies have shown its beneficial effects on histopathological changes, suggesting a potential use in the treatment of inflammatory conditions [81]. One of the most studied effects of menthol is its analgesic effect. Topically applied menthol or peppermint oil has been shown to have analgesic properties, making them effective in muscle pain relief and post-exercise recovery. Gillis et al. (2018) [82] studied the effect of menthol on recovery from exercise-induced muscle damage, while Topp et al. (2013) [83] analyzed the effect of menthol on blood flow and its potential impact on pain management. Menthol has also shown significant effects on the digestive system. Peppermint, which contains menthol as its main active ingredient, is used to treat gastrointestinal symptoms [84]. Similarly, menthol has a relaxing effect on the smooth muscles of the respiratory system, which may contribute to improved airflow in the airways. The findings suggest that menthol may be effective in relieving cold and cough symptoms [85]. Menthol also shows antibacterial and antifungal activity [81,86]. Menthol shows positive effects on alertness and cognitive function, improving concentration and attention. It may also support mental function under conditions of increased heat stress, making it potentially beneficial in situations requiring increased cognitive performance [87].

### 5.2. Biological Activities of Menthol Derivatives

**Figure 5:**
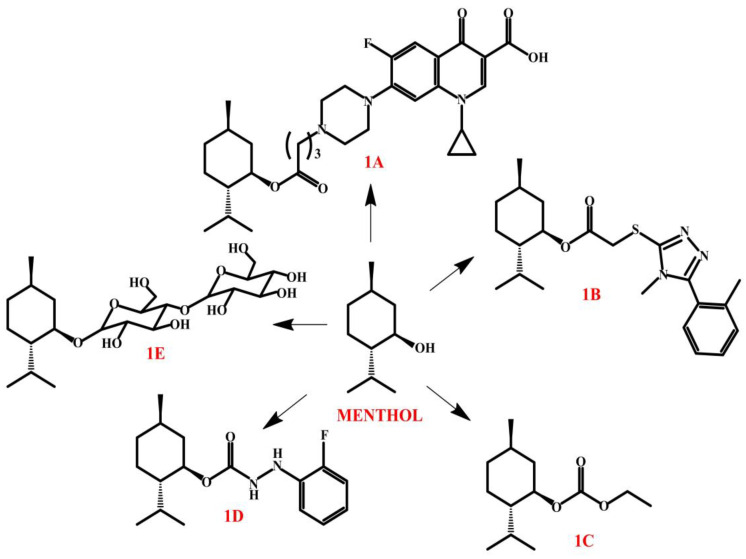
Menthol derivatives.

Menthol derivatives exhibit a broad spectrum of biological activities, including potent anti-inflammatory effects. Derivative **1A** (Figure 5) has proven to be particularly effective in this regard, exhibiting the ability to inhibit inflammatory mediators and reduce oxidative stress. Its mechanism of action is based on the modulation of inflammatory enzymes and a reduction in the production of pro-inflammatory cytokines, which may contribute to the reduction in inflammatory reactions in the body. With these properties, the derivative has potential applications in the treatment of autoimmune disorders and chronic inflammatory conditions, in which an excessive immune response leads to tissue damage [88]. Menthol derivative **1B** (Figure 5) showed the broadest spectrum and highest antifungal activity of the compounds tested. In bioassays, it effectively inhibited the growth of *Physalospora piricola*, *Colletotrichum orbiculare*, and *Fusarium oxysporum f.* sp. *cucumerinum*, outperforming the commercial fungicide chlorothalonil. Molecular docking studies showed that the mechanism of action of this derivative is based on interaction with cytochrome P450 14*α*-sterol demethylase (CYP51), suggesting its potential effect on ergosterol biosynthesis in fungal cells [89]. Derivative **1D** (Figure 5) has shown strong antifungal activity against *B. dothidea*, causing morphological damage to shoots and demonstrating high protective and curative efficacy in studies on apple fruit. In addition, **1D** showed strong activity as a laccase inhibitor, making it a promising candidate for further research into new fungicides [90].

Menthylmaltothioside (**1E**, Figure 5), a menthol derivative obtained by glycosidation, exhibits a broad spectrum of biological activities. Its antioxidant properties, confirmed by its ability to eliminate DPPH free radicals, are comparable to standard menthol, suggesting a potential use as an antioxidant. **1E** also shows antimicrobial activity, particularly against *Proteus* spp. and *Aspergillus flavus*, with efficacy similar to antibiotics such as ciprofloxacin and amphotericin B. In addition, tests on the beetle *Trogoderma granarium* confirmed its strong insecticidal properties due to its effects on the insect nervous system. The high efficacy of **1E** in these areas indicates its potential use as a natural antioxidant, antimicrobial, and insecticide [91]. Menthol derivatives show promising antiparasitic activity against *Trypanosoma cruzi*, *Leishmania braziliensis*, and *Plasmodium falciparum*. In in vitro studies, derivative **1C** (Figure 5) showed the highest selectivity and efficacy against these pathogens, indicating its therapeutic potential. In silico analyses confirmed that menthol derivatives stably bind to the dihydroorotase dehydrogenase (DHODH) active site, suggesting their mechanism of action as inhibitors of this metabolic pathway. In addition, their favorable pharmacokinetic properties indicate their potential use as oral drugs [92].

## 6. *β*-Pinene

### 6.1. Biological Activities

The literature provides a wide spectrum of information about each of the pinene isomers (*α*-, *β*-, and γ-pinene). The high interest in these compounds is linked to their significant biological activities, high bioavailability, and high natural distribution. The sources of these monoterpenes are *Piper nigrum*, *Juniperus species*, *Cannabis sativa* L., *Cedrus species*, *Rosmarinus officinalis* L., and many more [93,94]. It is commonly known that the biological activities of compounds are associated with their isomers and enantiomeric forms. *α*-Pinene is frequently described as having antimicrobial, antifungal, antioxidative, anti-leishmanial, and neuroprotective activities, with its effects depending on the specific enantiomers present [95,96,97]. In the case of *β*-pinene, the literature also highlights its antimicrobial, antiviral, anticancer, antioxidative, and anti-inflammatory activities [94,95]. These compounds often serve as the basis for the synthesis of new derivatives, exhibiting diverse biological activities that surpass those of their precursors.

### 6.2. Biological Activities of β-Pinene Derivatives

**Figure 6:**
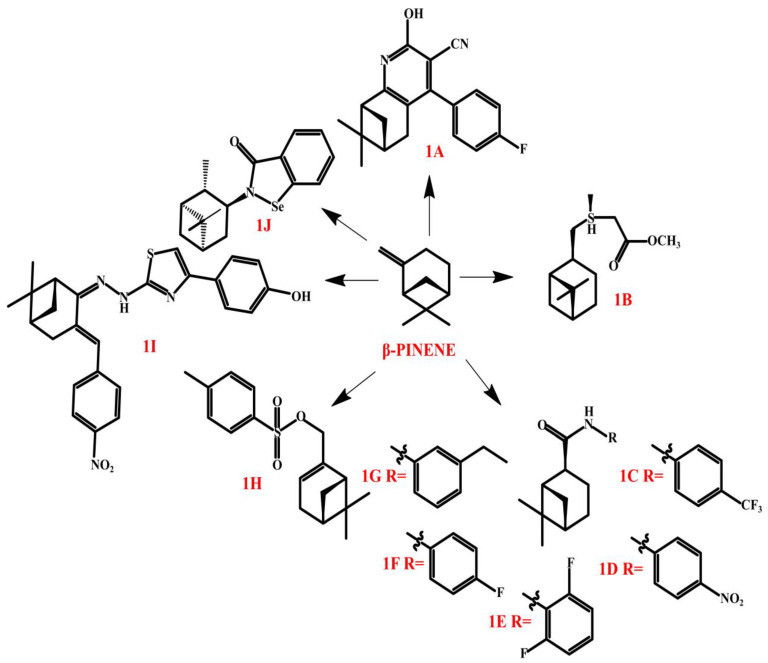
*β*-pinene’s derivatives.

There is a wide spectrum of pinenes derivatives, which are studied for various biological activities. The compounds presented by Liao et al., who focused on obtaining 3-cyanopyridine derivatives of (−)-*β*-pinene (**1A**, Figure 6), are interesting examples of monoterpene derivatives studied for their antimicrobial and antifungal activities [98]. The authors indicated that the evaluated compounds showed moderate activity against *S. aureus* and *S. epidermidis*, weak activity against *K. pneumoniae* and *E. aerogenes*, and moderate activity against *C. albicans*. Remaining on the subject of antibacterial and antifungal activity, it is worth mentioning the work presented by Jin et al. [99], who indicated antifungal activity against various pathogenic fungi recorded for hydronopyl quaternary ammonium salts from *β*-pinene. Antifungal activity tests demonstrated that at 500 mg/L, all compounds exhibited some level of inhibition against five phytopathogenic fungi. Notably, two compounds showed 100% inhibition against *Phoma citricarpa* and *Pestalotiopsis actinidia*, surpassing the effectiveness of carbendazim [100]. Activity against *Penicillium tardum* has been observed in the case of sulfur-containing (1S)-(−)-*β*-pinene derivatives (**1B**, Figure 6) [101].

An interesting transformation of *β*-pinene was conducted by Shi et al. [102], who proposed a series of monoterpene-based derivatives containing amide moieties and acylthiourea moieties (**1C**–**1G**, Figure 6). The analyses were performed against *Colletotrichum gloeosporioides*, *Fusarium proliferatum*, *Alternaria kikuchiana*, *Phomopsis* sp., and *Phytophthora capsici*. The most active compounds turned out to be **1C**–**1F,** which revealed a wide spectrum of activity, whereas **1G** was active against *Phytophthora capsici*. Scientists underlined the significance of fluorine atoms and nitro groups, as well as trifluoromethyl groups, on the benzene ring of the new compounds for antifungal properties (*Colletotrichum gloeosporioides*, *Fusarium proliferatum*, *Alternaria kikuchiana*, *Phomopsis* sp.), whereas strong anti-*Phytophthora capsici* activity was linked with ethyl groups at the meta-position on the benzene ring.

Another significant modification of *β*-pinene was performed by Nikitina et al. [103], who focused on pinene sulfide. Detail analysis revealed that pinene sulfide (methyl [(1R,2R,5R)-(6,6-dimethylbicyclo[3.1.1]hept-2-yl)-methylthio]ethanoate) was able to inhibit platelet receptors. This compound suppressed both platelet activity and coagulation hemostasis, making it a potential candidate for developing innovative anti-aggregation medications and enhancing the stability of blood products.

Derivatives of pinene were also synthesized in order to obtain antitumor agents. An example is (6,6-dimethylbicyclo[3.1.1]hept-2-en-2-yl)methyl-4-methylbenzenesulfonate) (**1H**, Figure 6), presented by Ye et al. [104]. Previous analyses [104] revealed that the compound was able to inhibit hepatoma carcinoma cell BEL-7402. The findings indicated that **1H** exhibited significant anti-liver cancer activity in vitro, with an IC**_50_** of 84.7 μmol/L. In vivo, it suppressed tumor growth in a dose-dependent manner. Additionally, **1H** halted hepatoma cell proliferation at the S phase, triggered apoptosis, and reduced the expression of C-myc, CDK2, and CyclinE, while increasing p53 levels [104].

A series of *β*-pinene-based thiazole derivatives synthesized by Wang et al. [105] was studied in detail for antitumor activity. The most promising turned out to be compound **1I,** presented in Figure 6, which displayed high cytotoxic activity against Hela, CT-26, and SMMC-7721 cell lines. Detail studies confirmed that the compound acts using reactive oxygen species, leading to mitochondrial dysfunction signaling pathways.

Antioxidant and antitumor activity were also found in the newly synthesized pinene derivatives by Obieziurska et al. [106]. Benzisoselenazol-3(2H)-ones with nitrogen-substituted monoterpenes groups—p-menthane, pinane, and carane—were successfully synthesized. N-isopinocampheyl-1,2-benzisoselenazol-3(2H)-one (**1J**, Figure 6) exhibited the strongest peroxide scavenging ability and the highest antiproliferative activity against the human promyelocytic leukemia HL-60 cell line. Meanwhile, N-menthyl-1,2-benzisoselenazol-3(2H)-one showed the greatest anticancer potential against the MCF-7 breast cancer cell line. The relationship between the structure, chirality, and biological activity of the synthesized organoselenium compounds has been extensively analyzed.

## 7. Thymol

### 7.1. Biological Activity

Thymol has a wide spectrum of biological activity, which makes it a promising compound in various medical and therapeutic applications. It has strong antibacterial properties, especially against Gram-positive and Gram-negative bacteria, and its effectiveness can be increased by using nanoformulations that improve the solubility and bioavailability of the compound [107,108,109]. In addition to its antibacterial activity, thymol also has antifungal activity, which means it can be used as a means of combating fungal infections and protecting plants against pathogens [109,110]. An important aspect of thymol’s action is its antioxidant properties, resulting from its ability to neutralize free radicals and protect lipids from peroxidation, which may contribute to protecting the body against oxidative stress and degenerative diseases [111,112,113]. In turn, its anti-inflammatory activity is associated with the inhibition of ERK1/2 and NF-κB signaling pathways and the reduction in pro-inflammatory cytokine expression, which may be used in the therapy of inflammatory diseases [114]. More and more studies also indicate the potential anticancer activity of thymol and its derivatives. Their mechanism of action is based on the induction of apoptosis and DNA damage in cancer cells, which makes them interesting candidates for further research in the field of cancer therapy [115,116]. In addition, thymol shows antidiabetic activity, acting as an inhibitor of glucosidase and amylase enzymes, which may contribute to the regulation of blood sugar levels and provide potential support in the treatment of diabetes [117]. In addition, thymol has an immunomodulatory effect, affecting the immune system by increasing the number of leukocytes and modulating the humoral response, which may be important in improving the body’s immunity and supporting the treatment of autoimmune diseases [118]. To sum up, thymol is a compound with multifaceted action, and its diverse biological properties give it a wide potential for use in medicine, health care, and agriculture.

### 7.2. Biological Activities of Thymol Derivatives

**Figure 7:**
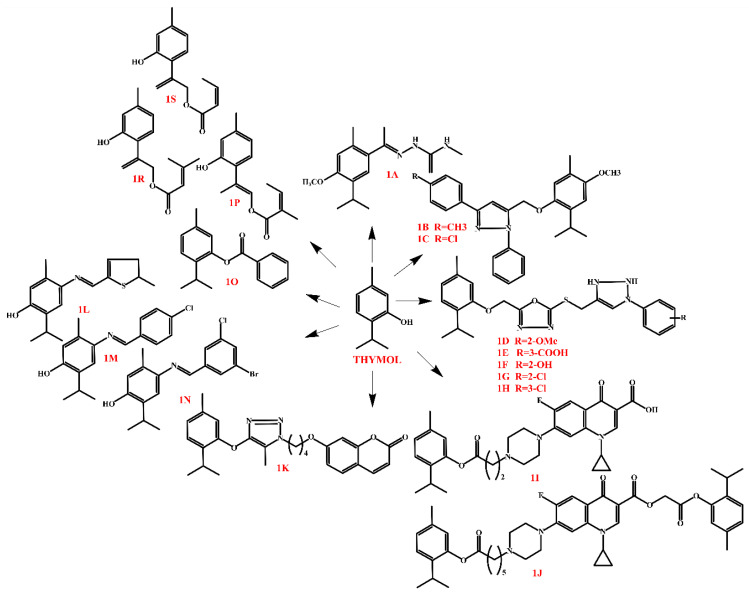
Thymol’s derivatives.

Thymol derivatives exhibit a wide range of biological activities, including anticancer, antibacterial, antifungal, and insecticidal effects. The structural modifications of thymol, such as thiosemicarbazone, coumarin, pyrrole, methoxy-pyrazole, and halogen substitutions, significantly increase its efficacy, making these compounds promising candidates for further pharmacological and agrochemical studies. In terms of anticancer activity, thymol derivatives exhibit the ability to inhibit cancer cell proliferation by inducing apoptosis and arresting the cell cycle. Derivative **1A** (Figure 7), a hybrid of thiosemicarbazone and thymol, showed the ability to induce apoptosis and arrest the cell cycle in the G2/M phase. In turn, derivatives **1B** and **1C** (Figure 7), containing a p-methoxy-pyrazole group, showed better cytotoxicity, suggesting that the substitution of the ether group improves their anticancer activity. Derivatives **1D**–**1H** (Figure 7) showed selectivity for specific tumor lines, with 6d being particularly effective against HCT-116. Thymol–ciprofloxacin hybrids (**1I** and **1J**, Figure 7) showed some activity, although no clear trend in the structure–activity relationship was demonstrated. Derivatives **1K** (Figure 7), thymol–coumarin hybrids, showed good activity by inducing apoptosis, while derivatives **1L**–**1N** (Figure 7), containing halogen groups, were characterized by high antitumor efficacy [119,120,121,122].

In terms of antibacterial activity, the most active derivatives are **1O**–**1S** (Figure 7). Derivative **1O**, a hybrid of thymol and benzoic acid, was characterized by the highest antibacterial activity, which indicates a beneficial effect of the presence of this group. Derivatives **1P**–**1S**, which are hybrids of ursolic acid and thymol, also showed good efficacy against selected bacterial strains, although their activity was weaker compared to ursolic acid [117,118,119].

Studies on antifungal activity have shown that thymol derivatives can be effective plant protection agents against fungal infections. This series of compounds was based on thymol derivatives through the incorporation of sulfonamide moieties and the addition of various other groups. The derivatives were particularly effective against *Phytophthora capsici*, outperforming commercially used fungicides such as azoxystrobin and carbendazim. One of the derivatives showed the highest activity against *Sclerotinia sclerotiorum*, both in vitro and in vivo. Mechanistic studies have shown that the thymol derivative with a 2-naphthyl moiety caused shrinkage and disintegration of fungal hyphae, leading to the damage of cellular organelles and accumulation of antioxidant enzymes, which resulted in fungal cell death. The efficacy of the compound in biological tests exceeded 98% in curative and protective action at a concentration of 200 μg/mL, indicating its potential as an innovative fungicide with high efficacy [123].

Thymol derivatives also exhibit insecticidal and growth inhibitory effects. The most active compound among the tested derivatives was thymylbutanoate, which showed the strongest insecticidal effect against *Spodoptera exigua caterpillars*, being more than 15 times more toxic than thymol. In addition, thymylbutanoate effectively inhibited the activity of detoxification enzymes such as glutathione transferase (GST), carboxylesterase, and acetylcholinesterase (AChE), suggesting its potential role in eliminating insecticide resistance. Thymylbenzene and thymyl-3,4-methylenedioxycinnamate showed a strong inhibitory effect on the growth of *S. exigua* larvae, reducing their weight by more than half compared to the control group. Thymylbenzene had a particularly strong effect on larval development, making it a promising candidate for further research on natural plant protection products [124]. Thymol derivatives exhibit a wide range of biological activities, including anticancer, antibacterial, antifungal, and insecticidal properties. Structural modifications within thymol can significantly increase its efficacy, making these compounds promising candidates for further pharmacological and agrochemical studies.

## 8. Verbenone

### 8.1. Biological Activity

Verbenone, a bicyclic monoterpene belonging to the ketone group, can be found in numerous plants such as *Verbena triphvlla* and *Eucalyptus globulus* Labill [125]. Similarly to other secondary plant metabolites, the monoterpene reveals a wide spectrum of biological activities and is used in traditional medicine as well as industry. One of the most important is its antihyperglycemic activity, confirmed in in vitro studies which showed its ability to inhibit *α*-amylase and *α*-glucosidase enzymes. The activity was confirmed in an in vivo mice model [126]. Verbenone was also evaluated for its antifungal activity, which could be used in fungal infections. Studies on *Rosmarinus officinalis* L. essential oil, in which verbenone is a key component (11.10%), have shown significant antifungal activity against *Aspergillus flavus*. When tested at different concentrations (1, 1/2, and 1/4), the oil displayed stronger antifungal properties compared to the antibiotic gentamycin [127]. Essential oils rich in verbenone are characterized as good antibacterial agents against oral pathogens and as anti-inflammatory agents in carrageenan-induced pleurisy in mice [128]. The (1S)-(−)-verbenone enantiomer exhibited strong acaricidal effects against house dust mites and anticonvulsant activity in pentylenetetrazol-induced seizures in mice by modulating the RNA expression of neurotrophic and inflammatory factors [129]. Another essential oil, namely *Tagetes parryi* (containing 33.39% of verbenone), has revealed anti-inflammatory effects and has inhibited the production of pro-inflammatory mediators, nitric oxide, TNF-*α*, and IL-6, in LPS-stimulated macrophages [130].

### 8.2. Biological Activities of Verbenone Derivatives

**Figure 8:**
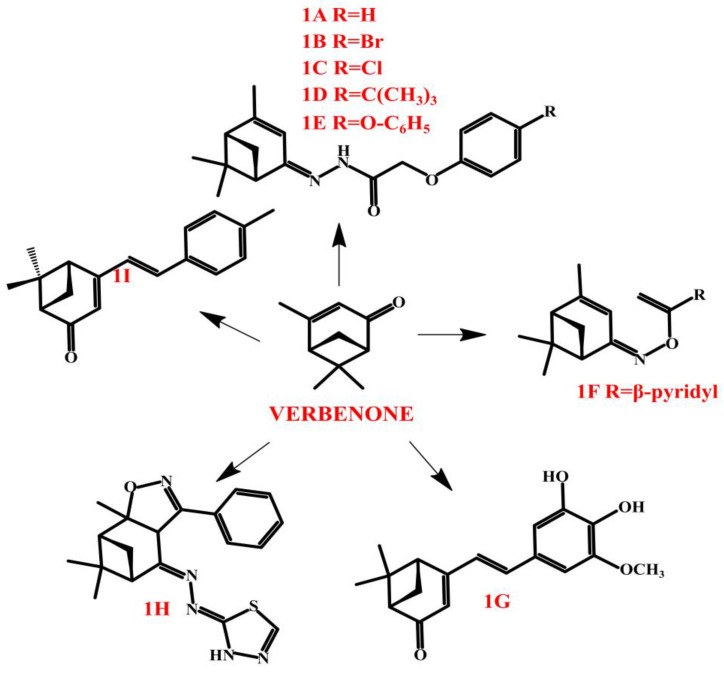
Verbenone derivatives.

Verbenone and its enantiomers are often used as molecular scaffolds for synthesizing novel active compounds. For example, (−)-verbenone hydrazones prepared by Nesterkina et al. [131]. The analgesic properties of hydrazones were evaluated through topical application in models of allyl isothiocyanate- and capsaicin-induced pain. The results suggest that verbenone hydrazones provide seizure protection both in the short term (6 h) and long term (24 h) by preventing chemically and electrically induced convulsions. Their strong analgesic effects may be linked to the binding of compounds **1A**–**1E** (Figure 8) to TRPA1/TRPV1 ion channels.

As previously mentioned in the ‘Biological activity’ Section, verbenone is characterized by strong antifungal activity. Many scientists focus on modifications of verbenone in order to improve its activity, which could be used in industry, and many of the attempts are successful. An example of a series of novel compounds are (Z)- and (E)-verbenone derivatives bearing an oxime ester moiety [128]. Among the twenty-seven compounds, the compound **1F** (Figure 8) demonstrated strong antifungal activity, inhibiting *Alternaria solani* (92.2%), *Physalospora piricola* (80.0%), and *Cercospora arachidicola* (76.3%) at 50 µg/mL. Its herbicidal properties were also preliminarily assessed. Seven compounds showed strong herbicidal activity, inhibiting *Brassica campestris* root growth by over 90% at 100 µg/mL and outperforming the commercial herbicide flumioxazin (63% inhibition) [125].

A significant modification has been provided by Mander at al. [132], who synthesized the (1S)-(−)-verbenone derivative, which has anti-breast cancer potential. The SP-8356, (1S,5 R)-4-(3,4-dihydroxy-5-methoxystyryl)-6,6-dimethylbicyclo[3.1.1]hept-3-en-2-one (compound **1G**, Figure 8), was recorded as an effective blocker of cell motility, which is presented as the most significant parameter of antimetastatic activity in cancer studies [133]. In regard to anti-breast cancer properties, **1G** treatment led to cell cycle arrest and inhibited growth in multiple breast cancer cell types with minimal cytotoxicity. Notably, it significantly decreased the motility and invasiveness of TNBC cells. Since NF-κB control genes are linked to epithelial–mesenchymal transition and metastasis, their suppression by **1G** plays a crucial role in restricting cancer progression.

The verbenone derivative has also been the basis of studies against liver cancer. Kim et al. [134] revealed that SP-8356 (**1G**, Figure 8) exhibited strong anti-proliferative effects on liver cancer cells by triggering apoptosis. Additionally, it reduced cell motility by modulating metastasis-related genes. Functional analysis indicated that these anticancer properties are linked to its suppression of the MAPK and NF-κB pathways.

Another potential anti-breast cancer compound is the isoxazoline-1,3,4-thiadiazole hybrid of (S)-verbenone (**1H**, Figure 8) [135]. One study focused on the determination of the cytotoxic and apoptotic effects in hormone-sensitive MCF-7 and triple-negative MDA-MB-231 breast cancer cells. These results highlighted the potential of (S)-verbenone isoxazoline-1,3,4-thiadiazole derivatives as promising agents for breast cancer therapy due to their strong apoptotic effects. The study emphasized their therapeutic potential in advancing breast cancer treatment by effectively inducing apoptosis.

Ju et al. [136] synthesized a novel antioxidant, a (1S)-(−)-verbenone derivative, namely [(1S,5R)-6,6-dimethyl-4-((E)-4-methylstyryl)bicyclo[3.1.1]hept-3-en-2-one] (**1I**, Figure 8), and examined its anti-ischemic effects. In rat neuronal/glial co-cultures, 1I reduced oxygen–glucose deprivation/reoxygenation (OGD/R)-induced neuronal damage and intracellular oxidative stress. Although its free radical scavenging ability was lower than trolox, it significantly increased astroglial heme oxygenase-1 (HO-1) expression. The HO-1 inhibitor tin protoporphyrin IX (SnPP) blocked its protective effects, suggesting that **1I** exerts neuroprotection through HO-1 upregulation in astroglial cells, making it a potential therapeutic target for ischemic stroke.

## 9. Menthone

### 9.1. Biological Activity

Menthone (2-Isopropyl-5-methylcyclohexanone) is a widespread monoterpene with a fresh smell characteristic of peppermint (*Mentha x piperita*) [137]. It is known that the compound reveals numerous valuable biological activities which, along with the characteristic smell, are often used in the industry. Menthone has been studied both in vitro and in vivo in detail, which allowed the confirmation of its antioxidant, neuroprotective, anti-inflammatory, and anti-viral activities [138]. In vitro Ellman’s assay revealed AChE inhibitory activity, which was confirmed by molecular docking [139]. Additionally, the monoterpene is able to carry out Fe(III) and Cu(II) reduction, as well as Fe(II) chelation, which are significant in the context of neuroprotection [140]. Studies based on the asthmatic mice model revealed that menthone is an anti-inflammatory agent acting by decreasing eosinophils, NO, eotaxin, IL-4, IL-5, IL-1*β*, TNF-*α*, IL-10, and protein levels and increasing the IL-2, IFN-γ, and IL-6 levels in the BALF (bronchoalveolar lavage fluid) [137,141]. Many essential oils rich in this monoterpene reveal neuroprotective and antioxidant activity. An example is *Melissa officinalis* L., rich in menthone and isomenthone, whose pro-health properties were proven many times [142,143]. Mentha species characterized by high menthone content, exhibit various pro-health properties such as antimicrobial (i.e., against *Bacillus subtilis*, *Streptococcus aureus*, and *Pseudomonas aeruginosa*), cardioprotective, and antioxidant properties, along with low toxicity [144]. Additionally, the ability of MAPK (mitogen-activated protein kinase) modulation and PI3l/Akt pathways reveal good cytotoxicity potential [144].

### 9.2. Biological Activities of Menthone Derivatives

Due to its interesting biological activities, low molecular character, and good bioavailability, menthone is used as a molecular scaffold, but there is a limited number of studies presenting its active derivatives. The purpose of these reactions is to obtain highly active compounds in specific directions of treatment and synthesis involving menthone in order to offer great development opportunities.

**Figure 9:**
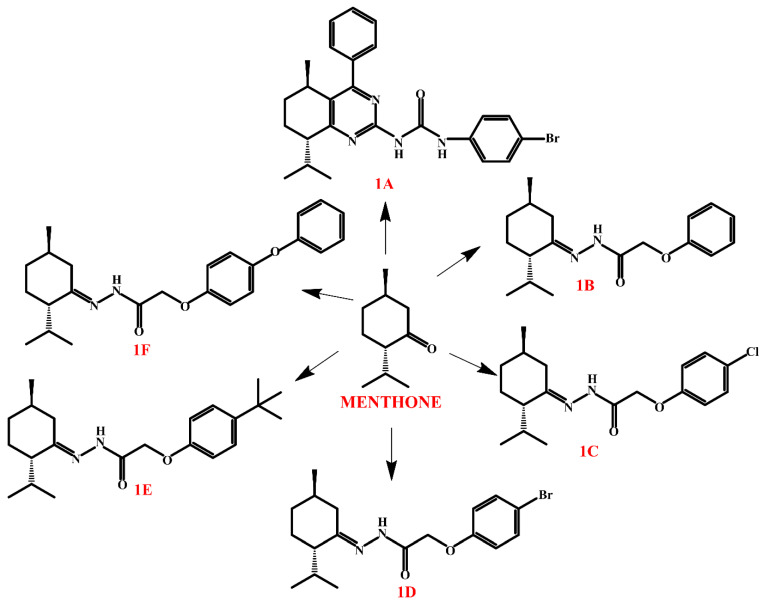
Menthone derivatives.

A good example is a series of newly designed menthone derivatives featuring pyrimidine and urea groups synthesized to investigate their potential as more effective natural product-based antitumor agents [145]. Detailed analysis revealed that the most probable mechanism of action of the most active compound (**1A**, Figure 9) is based on induction of cell apoptosis in HeLa cells and might arrest the cell cycle in the G2/M phase. Additionally, network pharmacology prediction and Western blot experiments showed that the compound can inhibit Hela cells by inhibition of the PI3K/Akt/mTOR pathway [145].

Nesterkina and co-workers presented menthone derivatives (the combination of menthone and phenoxyacetic acid) (compounds **1B**–**1F**, Figure 9) with probable anticonvulsant effect [146]. The properties of the compounds were assessed using the pentylenetetrazol (PTZ) model, which involves measuring the minimum effective doses (MEDs) of pentylenetetrazole required to trigger clonic–tonic convulsions (CTC) and tonic extension (TE) in experimental animals. Based on the experimental findings, it can be concluded that menthone hydrazones derived from para-substituted phenoxyacetic acids exhibit anticonvulsant activity in both PTZ and MES tests over short (3 h) and long (24 h) durations.

## 10. Conclusions

Molecular scaffolds play a pivotal role in new drug design. The synthesis of new compounds based on well-known naturally occurring structures allows us to obtain knowledge on more specific biological activities directed towards selected disorders such as cancer or neurodegeneration. The review paper focuses on the most often used monoterpenes, modifications to which allow us to obtain new structures with potential antibacterial, anticancer, anticonvulsant, and other activities. The most important biological activities achieved with the modification of monoterpenes’ structure are the following: carvacrol derivatives, with antibacterial, anticancer, enzyme-inhibiting, and potential glaucoma- and epilepsy-related applications; carvone derivatives, with antioxidant, antimicrobial, anticancer, anti-inflammatory, analgesic, and anticonvulsant applications; citral derivatives, with antifungal, anticancer, and anti-quorum sensing applications; menthol derivatives, with antiparasitic and enzyme-inhibiting applications; *β*-pinene derivatives, with antimicrobial, antifungal, and anticancer applications, as antiaggregation medications, enhancing the stability of blood products; thymol derivatives, with anticancer, antibacterial, antifungal, and insecticidal properties; verbenone derivatives, with seizure-protective, antifungal, and anti-breast and -liver cancer potential; and menthone derivatives, with antitumor and anticonvulsant applications.

In addition to the monoterpenes characterized in the paper, there are numerous interesting examples of secondary plant metabolites, which are often used as molecular scaffolds. Among them are limonene, camphor, thujone, borneol, linalool, and geraniol, the derivatives of which have been studied for oxidative stress, inflammation, and neurotoxicity reduction, GABA receptors, AChE inhibition, as well as increased stability and specificity towards neuronal targets of starting compounds.

The structural adaptability of monoterpenes, particularly their chirality, lipophilicity, and functional groups, makes them attractive candidates for drug discovery. The growing interest in monoterpenes as molecular scaffolds highlights their significant role in green chemistry and sustainable drug development, offering innovative solutions for future pharmacological applications.

## Data Availability

The research data are available from the authors.
